# Analyse sérologique de la toxoplasmose pergravidique: évaluation des risques et perspectives du dépistage prénatal au centre hospitalier universitaire de Bobo Dioulasso au Burkina Faso

**Published:** 2012-06-22

**Authors:** Sanata Bamba, Der Adolphe Some, Cathy Chemla, Régine Geers, Tinga Robert Guiguemde, Isabelle Villena

**Affiliations:** 1 Universitaire Sanou Souro de Bobo Dioulasso, Burkina Faso; 2 Laboratoire de Parasitologie-Mycologie, Centre National de Référence de la Toxoplasmose, CHU Maison Blanche, Reims, France; 3 Laboratoire de Parasitologie-Mycologie, Institut supérieur des Sciences de la Santé / Université polytechnique de Bobo Dioulasso, Burkina Faso

**Keywords:** Toxoplasmose, sérodiagnostic, séroconversion, grossesse, risques, evaluation, dépistage

## Abstract

**Introduction:**

La présente étude rapporte les données sérologiques de 306 sérums collectés chez des parturientes au CHU de Bobo Dioulasso et analysés rétrospectivement au CHU de Reims en 2011. Le but était de déterminer le statut sérologique de ces parturientes et d'en déduire la conduite à tenir.

**Méthodes:**

La recherche des IgG et des IgM anti toxoplasmiques était systématique. Les techniques d'agglutination haute sensibilisée et celle d'Immunocapture M ont servi à la recherche respective des anticorps spécifiques IgG et des IgM.

**Résultats:**

Sur 306 sérums analysés, 95 (31%) avaient des IgG positifs et aucun n'avait des IgM. Deux cent onze (211) sérums (69%) des sérums n'avaient ni IgG, ni IgM.

**Conclusion:**

Nos résultats montrent que 31% des femmes en dehors d'une immunodépression sous jacente, possèdent une immunité résiduelle vis à vis de Toxoplasma gondii et n'ont pas la nécessité d'avoir une surveillance sérologique pendant la grossesse. Cependant, 69% (211) des parturientes sont à risque d'une séroconversion, et devraient bénéficier de conseils hygiéno diététiques, associés à une surveillance sérologique durant la grossesse. Ces résultats montrent l'intérêt de mettre en place des mesures de prévention contre la toxoplasmose congénitale, étant l'une des affections materno - foetales les plus fréquentes par la mise en place d'un diagnostic prénatal de la toxoplasmose en routine dans notre hôpital.

## Introduction

Toxoplasma gondii (toxoplasme) est un protozoaire intracellulaire obligatoire capable de parasiter presque toutes les cellules des animaux à sang chaud. Le cycle parasitaire comporte un cycle sexué chez l'hôte définitif (chats et autres félidés) et un cycle asexué chez l'hôte intermédiaire (homéothermes) [1]. L'homme peut se contaminer en consommant des produits souillés par des oocystes, comme des végétaux (légumes, fruits) mais aussi de l'eau et de la viande contenant des kystes [2, 3]. Chez la femme enceinte, une primo-infection peut être à l'origine d'une toxoplasmose congénitale. La positivation (d'une valeur négative à une valeur positive) en IgG et IgM (avec la même technique et dans la même série) est la preuve de cette primo-infection ou d'une séroconversion toxoplasmique [4, 5]. La datation de la primo-infection chez la femme enceinte doit être réalisée. Au cours de la grossesse, la sérologie toxoplasmose doit être répétée de façon régulière dès lors que le premier examen est négatif. En cas de séroconversion en cous de grossesse, le risque d′infection foetale croît régulièrement du début à la fin de la grossesse, au contraire de la gravité qui diminue au fur et à mesure, les séquelles foetales étant plus importantes lors d′infections précoces [3–5]. La toxoplasmose congénitale (TC) est potentiellement mortelle pour le foetus. Le diagnostic clinique de TC associe classiquement rétinochoroïdite, hydrocéphalie et calcifications intracrâniennes, mais tous les organes peuvent être atteints [3–5]. Le dépistage de la toxoplasmose congénitale implique la surveillance maternelle en cours de grossesse. Les analyses consistent à doser les anticorps (IgG et IgM) par deux techniques différentes.

Ces techniques reposent sur l'immuno fluorescence et surtout sur des méthodes ELISA (Enzyme-Linked Immunosorbent Assay) pour la détection des IgG et des IgM. Les techniques d'imunocapture ICT ou ISAGA (Immuno-Sorbent-Agglutination Assay) permettent de déceler encore des IgM spécifiques plusieurs mois après la contamination. La détection des IgA et des IgE est réalisée par certains laboratoires spécialisés dans des cas bien ciblés où la datation s'avère difficile [4, 6, 7]. Mais, l'interprétation d'une sérologie toxoplasmique reste parfois délicate, notamment chez la femme enceinte en fonction du terme de la grossesse au moment de la réalisation du prélèvement avec parfois la difficulté d'exclure une contamination périconceptionnelle lorsque le profil sérologique associe des IgG à des IgM anti-toxoplasmiques voire des IgA spécifiques [7]. Chez la femme enceinte, des études rapportent des séroprévalences supérieures à 60 % en Afrique [8]. Au Burkina Faso, les auteurs notifient des prévalences globales de 28,3% (études de 2006) [9]et de 29,1%(études de 2009) chez les parturientes [10].

Le présent travail rapporte les données d'une analyse rétrospective du sérodiagnostic de la toxoplasmose chez les femmes enceintes sans notion de résultats sérologiques antérieurs. L'objectif de notre étude était de déterminer le statut sérologique initial des parturientes et d'en déduire la conduite à tenir.

## Méthodes

### Type, période et site d’étude

La présente étude a concerné les parturientes admises à la maternité de l′hôpital universitaire de la ville de Bobo Dioulasso au Burkina Faso de Janvier à Juillet 2011. Elle est située à l′ouest du Burkina Faso et compte environ 1,7 millions d′habitants. L′hôpital universitaire de Bobo-Dioulasso est un centre de référence pour quatre des 13 régions sanitaires du pays [11]. Dans cet hôpital, comme ailleurs au Burkina Faso, le diagnostic de la toxoplasmose ne rentre pas dans le bilan prénatal. Il n'y existe aucun programme ni de dépistage ni de suivi de la toxoplasmose chez les parturientes. La présente étude a été approuvée par le Comité national d′éthique avant l′exécution. Tous les participants ont signé un formulaire de consentement libre et éclairé.

### Echantillonnage

Les sérums ont été collectés chez les femmes enceintes d'au moins 2 mois sans notion de résultats sérologiques antérieurs et conservés à -20°C. Ils ont été analysés rétrospectivement au laboratoire de Parasitologie-Mycologie du CHU de Reims en France.

### Diagnostic sérologique

Tous les sérums de cette étude ont été analysés par les techniques de sérologie utilisées au laboratoire de Parasitologie Mycologie du CHU de Reims en France [7, 12–14]. Les IgG spécifiques de T. gondii ont été titrées en U/mL par la technique d'Agglutination Directe Haute Sensibilité (ADHS). Le principe du test repose sur l'agglutination de toxoplasmes formolés (antigène) par des anticorps (IgG) spécifiques présents dans le sérum à analyser dans des plaques de micro tritration. L'antigène est préparé selon le protocole de Desmonts et Remington. Tous les échantillons (traités préalablement avec le dithiotréitol) sont dilués de deux en deux et mis en présence d′une suspension de tachyzoïtes de Toxoplasma gondii pour la détection des IgG spécifiques. Les résultats ou les titres sont exprimés en unité par millilitre (U/mL). Si le sérum contient des anticorps anti-toxoplasmiques, les toxoplasmes s'agglutinent sous la forme d'un voile. Dans le cas contraire, les toxoplasmes sédimentent au fond des plaques. La réaction est lue visuellement. La valeur seuil de la technique est un titre de 6 U / ml) [14]. Les IgM ont été recherchées par la technique d'immunocapture (IC-T) mise au point par le CHU de Reims en France. Le sérum à tester est mis en contact des plaques de microtitration (constituées de barrettes sécables) préalablement sensibilisées par un anticorps monoclonal anti-IgM humain pour la recherche des IgM. Chaque échantillon de sérum est dilué (1 / 100) et déposé dans trois puits adjacents. Les plaques sont ensuite incubées à 37 °C pendant 2 h. Après le lavage, une suspension formolée de tachyzoïtes (obtenus par le laboratoire de Parasitologie-Mycologie du CHU de Reims à partir de la souche RH de Toxoplasma gondii et traités à la trypsine) correspondant à l'antigène, est ajoutée à des volumes de 100 µL, 150 µL et 200 µL respectivement dans les trois puits. Après 18 h d'incubation, la réaction anticorps-antigène se traduit par une agglutination qui est la sédimentation complète (score de 0), tandis qu'un voile complet sur toute la cupule est considérée comme une valeur très positive (score de 4). La valeur cumulative des trois puits pour un échantillon donné peut donc varier de 0 à 12. La lecture est faite par une méthode automatisée à partir d'un lecteur de microplaques (MR 7000; Dynatech, Guyarcourt, France) couplé à un logiciel approprié (Orkys) [8, 12–14].

### Analyse des données

Le logiciel EpiData version 3.1 a été utilisé pour la saisie et l′analyse des données.

### Aspects éthiques

La présente étude a été approuvée par le Comité national d′éthique avant l′exécution. Toutes les participantes ont signé un formulaire de consentement libre et éclairé.

## Résultats

### Données socio-démographiques

Dans la présente étude rétrospective, 306 sérums ont été collectés chez des femmes enceintes d'au moins deux mois et sur toute la période de la grossesse. Sur les 306 sérums analysés, 21,4% (n= 65) ont été prélevés au cours du premier trimestre de la grossesse vs 33,9% (n= 104) et 44,7% (n= 137) pour respectivement deuxième et troisième de la grossesse (Table 1). La moyenne d’âge des femmes enceintes était de 26,9 ans (extrêmes: 17 à 47 ans).

**Table 1:** Prévalence des anticorps anti Toxoplasma gondii selon le trimestre de la grossesse chez les 306 femmes testées

Anticorps anti *Toxoplasma gondii*	1 ^er^ Trimestre		2 ^ème^ Trimestre		3 ^ème^ trimestre		Total	
	Effectif	%	Effectif	%	Effectif	%	Effectif	%
IgG négatif/ IgM négatif	33	50,8%	79	75,9%	99	72,3%	211	69%
IgG positif/IgM négatif	32	49,2%	25	24,1%	38	27,7%	95	31%
Total des cas	65	21,4%	104	33,9%	137	44,7%	306	100%

### Données de l’étude sérologique

Pour les 306 sérums analysés, 31% (n = 95) avaient des IgG en ADHS et aucun sérum n'avait d'IgM en technique d'immunocapture. La séroprévalence globale était donc de 31% (95/306) (Table 1). Au total 69% (211/306) des sérums analysés n'avaient ni IgG ni IgM (Table 1). Sur les 211 sérums négatifs testés, 50,8%(33/65) étaient collectés au premier trimestre Vs 75,9% (79/104) et 72,3%(99/137) pour le deuxième et troisième de la grossesse (Table 1).

## Discussion

La présente étude a été conduite à Bobo Dioulasso chez les femmes enceintes sans notion de résultats sérologiques antérieurs. L'objectif de notre étude était de déterminer le statut sérologique initial des parturientes et d'en déduire la conduite à tenir. Les résultats indiquent une séroprévalence toxoplasmique globale de 31% définie par la présence des IgG anti-toxoplasmiques (Table 1). Nos résultats corroborent ceux retrouvés au Burkina Faso sur deux études préliminaires en 2006 par Simporé et al [9] et en 2009 par Ouermi et al [10] chez les femmes enceintes. A partir de ces trois études faites au Burkina Faso, il apparaît nécessaire de mettre en place un programme national de dépistage et de surveillance de la toxoplasmose chez les femmes enceintes au Burkina Faso.

Si l'on compare la séroprévalence obtenue dans la présente étude à celle retrouvée dans différents pays africains (Table 2), il s'avère que la prévalence de la toxoplasmose au cours de la grossesse varie selon les pays, en Cote d'Ivoire, au Nigeria, au Gabon, en République Centre Africaine et au Maroc. Ainsi, en Cote d'Ivoire, Adou-bryn et al retrouvaient un taux de séroprévalence à 60% [15], alors qu'au Nigeria, Olusi et al [16], notifiaient une prévalence de 43,7% [16], Ndiaye et al enregistraient 34,5% de taux de séroprévalence toxoplasmique au Sénégal [17]. En République Centre Africaine, Morvan et al [18] notifiaient 60% de taux de prévalence alors qu'au Gabon, Mpiga et al [19] notifiaient un taux de séroprévalence de 56%. Au Maroc, El Mansouri et al [20] décrivaient une séroprévalence toxoplasmique de 50,6%.

**Table 2:** Données comparatives sur la séroprévalence de la toxoplasmose au cours de la grossesse dans différents pays africains

Pays	Effectif de femmes enceintes analysées	Année	Auteurs	Technique de sérologie	Séroprévalence de la Toxoplasmose
**Côte d'Ivoire**	1025	2001	Adou-bryn et *al*	IFI	60%
**Nigeria**	606	1996	Olusi et al	ELISA	43,7%
**Sénégal**	941	2006	Ndiaye D et *al*	ELISA	34,5%
RCA*	1953	1998	Morvan et *al*Mpiga	ELISA	50,6%
**Gabon**	839	2009	et al	ELISA	56%
**Maroc**	2456	2007	El Mansouri et *al*	ELISA	50,6%

* République Centre Africaine

Dans les pays européens comme en France, des auteurs rapportaient une séroprévalence toxoplasmique moyenne de 43% [21] alors qu'en Italie, De Paschale et al [22] notifiaient 28,3% de séroprévalence toxoplasmique au cours de la grossesse. En Thaïlande, Nissapaton et al [23] enregistraient une prévalence toxoplasmique de 29,1% chez les femmes enceintes. Au Brésil, dans le continent sud américain, Barbosa et al [24] enregistraient un taux de prévalence de 66, 3% au cours de la grossesse. Toutefois, ces variations de la séroprévalence selon les régions et les pays pourraient être liées aux facteurs géo-climatiques (pluviométrie, les températures), sachant que la chaleur et l'humidité sont des facteurs favorisant la conservation des oocystes dans le sol et participent ainsi au maintien d'une prévalence élevée. On sait aussi que cette différence de prévalence peut également être liée à la divergence des habitudes alimentaires [2, 3, 5].

Dans la présente étude, il est à noter qu'aucun des sérums testés n'avait d'IgM (Table 1). Cette tendance est différente de celles publiées antérieurement pour le Burkina Faso [9, 10], l'Italie [22] et la Thaïlande [23] où à des IgM étaient associées à la présence des IgG dans respectivement 4,7%, 1,7% et 6,7% des cas. Toutefois, la présence d'IgG et l'absence d'IgM (IgG+ et IgM-) dans notre série pour une première sérologie toxoplasmique sans aucune antériorité chez la femme enceinte, plaide pour une immunité ancienne probable. Dans ce contexte, 31% des femmes de notre étude ne sont pas susceptibles de transmettre l'infection à leur foetus en dehors de toute immunodépression.

Aussi, elles ne nécessiteraient aucune surveillance complémentaire au cours de la grossesse si un programme de dépistage et de suivi de la toxoplasmose pergravidique était mis en place [4–6]. Cependant, un deuxième sérum, généralement recommandé 3 semaines après le premier sérodiagnostic, permettrait de suivre l’évolution du titre des IgG. Ainsi, un titre stable permettrait de conclure définitivement à une infection ancienne. Cependant, si une augmentation du titre des anticorps était observée, elle devrait conduire à une datation plus précise de l'infection avec l'aide des techniques sérologiques complémentaires à associer pour évaluer le risque encouru par le foetus. Mais une élévation isolée du titre des IgG peut aussi être la conséquence d'une réactivation sérologique du fait d'une infection ancienne sans incidence pour le foetus, en dehors de tout contexte d'immunodépression et l'intérêt de la recherche d'autres marqueurs d’évolutivité peut également être suggéré.

Cependant, en cas d'immunodépression connue, la présence d'IgG et l'absence d'IgM même si elle évoque une infection ancienne probable, peut nécessiter une surveillance sérologique et doit être interprété en fonction de la clinique et du degré d'immunodépression. Somme toute, seule, l′analyse en parallèle de 2 sérums prélevés à distance (3 semaines), dans le même laboratoire, par la même technique et dans la même série, permet une conclusion définitive, ce qui n'est pas le cas dans la présente étude rétrospective [4–6]. Deux cent onze (69%) femmes de l’étude n'ont ni IgG, ni IgM spécifiques. Ceci témoigne d'une absence d'immunité toxoplasmique avec un risque de seroconversion per gestationnelle [4–6]. Ces femmes doivent être contrôlées sérologiquement de façon régulière pour dépister une éventuelle seroconversion (apparition d'IgM puis d'IgG). Le risque potentiel de séroconversion per gestationnelle dans notre étude concerne 50,7%, 75,9% et 72,3% des parturientes pour respectivement le premier, deuxième et troisième trimestre de la grossesse (Table 1).

En cas de séroconversion avérée, le foetus est à risque d'infection congénitale, risque qui varie en fonction de l’âge de la grossesse au moment de la contamination maternelle. Les parturientes concernées devraient entrer dans un programme rigoureux de surveillance (échographie et dépistage biologique anténatal). Il est à noter que des cas de séroconversion anteconceptionnelle avec des marqueurs d’évolutivité et de signes cliniques (adénopathies) peuvent s'accompagner d'une transmission foetale [4–6]. Ceci a été décrit de façon très exceptionnelle. Dans la présente étude, la proportion des parturientes qui sont encore à risque de séroconversion en fin de grossesse car non immunisées (72,3%) est non négligeable. Une limite dans notre étude concerne les cas où cette unique sérologie a été effectuée au troisième trimestre de la grossesse et est positive. En effet, il devient alors beaucoup plus difficile d'exclure de façon formelle une survenue de séroconversion en début de grossesse associée à des IgM fugaces qui aurait alors disparu pour 27,7% (38/137) des cas de sérologie positive au troisième trimestre de grossesse. Cependant, une étude prospective avec un suivi sérologique précoce permettrait de conclure définitivement la présente hypothèse.

Afin de réduire le risque de transmission de la toxoplasmose congénitale chez les 211 femmes non immunisées de notre étude, deux attitudes préventives pourraient être recommandées pendant la grossesse: sérologies régulières si possible mensuelles jusqu’à l'accouchement et même en post partum proche et l'application des recommandations hygiéno diététiques.

## Conclusion

La toxoplasmose est une infection pouvant avoir des conséquences graves, voire fatales chez le foetus en cas de séroconversion chez les parturientes et l'absence d'une prise en charge précoce et adéquate.

La séroprévalence globale de la toxoplasmose chez les parturientes dans notre étude est de 31%. Ces données montrent que 69% des femmes enceintes sont susceptibles de se contaminer pendant la grossesse du fait de leur absence d'immunité antitoxoplasmqiue avec alors un risque de toxoplasmose congénitale pour le foetus.

La contamination maternelle sera matérialisée par l'apparition d'une séroconversion. Sur la base de nos résultats, il serait souhaitable de mettre en place un programme de surveillance sérologique le plutôt possible pendant la grossesse comme première mesure de prévention de la toxoplasmose congénitale. Celui-ci doit être associé à une information claire des femmes enceintes non immunisées vis-à-vis de la toxoplasmose proposant des recommandations prophylactiques (règles hygiéno-diététiques, d'hygiène, éviter le contact avec les chats et les activités du jardinage). Toutefois, seule une étude prospective permettrait d’évaluer réellement le risque de séroconversion, de connaitre le nombre de cas de toxoplasmose congénitale et de mettre en place la prise en charge des enfants concernés dans la cadre d'un programme national de surveillance de la toxoplasmose au CHU de Bobo Dioulasso.

## Remerciements

Nos sincères remerciements s'adressent à l'agence universitaire de la Francophonie, à tout le personnel de la maternité et du laboratoire de parasitologie du CHU de Bobo Dioulasso (Burkina Faso), à toute l’équipe du laboratoire de Parasitologie du CHU de Reims en France ainsi que toutes les femmes qui ont accepté de participer à l’étude.
